# Exosomes from primed MSCs can educate monocytes as a cellular therapy for hematopoietic acute radiation syndrome

**DOI:** 10.1186/s13287-021-02491-7

**Published:** 2021-08-18

**Authors:** Matthew H. Forsberg, John A. Kink, Anna S. Thickens, Bryson M. Lewis, Charlie J. Childs, Peiman Hematti, Christian M. Capitini

**Affiliations:** 1 Department of Pediatrics, University of Wisconsin School of Medicine and Public Health, 1111 Highland Ave, WIMR 4137, Madison, WI 53705 USA; 2 University of Wisconsin Carbone Cancer Center, Madison, WI USA; 3 Department of Medicine, University of Wisconsin School of Medicine and Public Health, 1111 Highland Ave, WIMR 4033, Madison, WI 53705 USA

**Keywords:** Mesenchymal stem cells, Exosomes, Monocytes, Lipopolysaccharide, Acute radiation syndrome, Hematopoiesis, IL-6

## Abstract

**Background:**

Acute radiation syndrome (ARS) is caused by acute exposure to ionizing radiation that damages multiple organ systems but especially the bone marrow (BM). We have previously shown that human macrophages educated with exosomes from human BM-derived mesenchymal stromal cells (MSCs) primed with lipopolysaccharide (LPS) prolonged survival in a xenogeneic lethal ARS model. The purpose of this study was to determine if exosomes from LPS-primed MSCs could directly educate human monocytes (LPS-EEMos) for the treatment of ARS.

**Methods:**

Human monocytes were educated by exosomes from LPS-primed MSCs and compared to monocytes educated by unprimed MSCs (EEMos) and uneducated monocytes to assess survival and clinical improvement in a xenogeneic mouse model of ARS. Changes in surface molecule expression of exosomes and monocytes after education were determined by flow cytometry, while gene expression was determined by qPCR. Irradiated human CD34+ hematopoietic stem cells (HSCs) were co-cultured with LPS-EEMos, EEMos, or uneducated monocytes to assess effects on HSC survival and proliferation.

**Results:**

LPS priming of MSCs led to the production of exosomes with increased expression of CD9, CD29, CD44, CD146, and MCSP. LPS-EEMos showed increases in gene expression of IL-6, IL-10, IL-15, IDO, and FGF-2 as compared to EEMos generated from unprimed MSCs. Generation of LPS-EEMos induced a lower percentage of CD14^+^ monocyte subsets that were CD16^+^, CD73^+^, CD86^+^, or CD206^+^ but a higher percentage of PD-L1^+^ cells. LPS-EEMos infused 4 h after lethal irradiation significantly prolonged survival, reducing clinical scores and weight loss as compared to controls. Complete blood counts from LPS-EEMo-treated mice showed enhanced hematopoietic recovery post-nadir. IL-6 receptor blockade completely abrogated the radioprotective survival benefit of LPS-EEMos in vivo in female NSG mice, but only loss of hematopoietic recovery was noted in male NSG mice. PD-1 blockade had no effect on survival. Furthermore, LPS-EEMos also showed benefits in vivo when administered 24 h, but not 48 h, after lethal irradiation. Co-culture of unprimed EEMos or LPS-EEMos with irradiated human CD34+ HSCs led to increased CD34+ proliferation and survival, suggesting hematopoietic recovery may be seen clinically.

**Conclusion:**

LPS-EEMos are a potential counter-measure for hematopoietic ARS, with a reduced biomanufacturing time that facilitates hematopoiesis.

## Introduction

Exposure to radiation from accidents or conflicts is a credible threat which poses a wide spectrum of challenges for the treatment and management of victims of radiation injury. Whole-body or significant partial-body exposure to ionizing radiation as low as 1 Gy can cause acute radiation syndrome (ARS) [1, 2]. ARS is a broad term used to describe a range of specific injuries to organ systems based on their sensitivities to different radiation doses. ARS can involve the hematopoietic and cutaneous system at > 1–3 Gy, the gastrointestinal system at 4–12 Gy, and the cerebro- or neurovascular systems at 10–20 Gy. Radiation doses at 4 Gy can cause death in 50% of people without supportive care [1].

The United States Department of Defense and Department of Health and Human Services define agents that protect against ARS into three categories depending on the time the agent is administered in respect to the radiation challenge [3]. Radio-protectors are agents administered before radiation. Radio-mitigators are delivered after exposure, but prior to the manifestation of tissue toxicity, attempting to prevent or attenuate the expression of radiation-induced side effects. No radio-protectors or radio-mitigators are currently approved by the Food and Drug Administration (FDA) for the prevention or treatment of ARS, although amifostine is approved to reduce the toxicity of radiation during cancer treatment [4]. A third category, radiotherapeutics, are also delivered after radiation exposure but should be able to treat radiation injury following tissue toxicity and function to cure or ameliorate ARS. While filgrastim and pegylated filgrastim are FDA approved as radiotherapeutics [5], a sub-group of radiotherapeutics in clinical testing consist of therapeutic cells. Cell therapies to date have mainly focused on mesenchymal stromal cells (MSCs), since they are pluripotent and theoretically could differentiate to replace injured cells [6]. PLX-R18 (Pluristem Therapeutics, Inc) is a placenta-derived, MSC product currently in testing in an open-label phase I study for the post-exposure prevention or treatment of hematopoietic ARS (NCT03797040) [7]. Other protective effects of MSCs may be related to their ability to secrete extracellular vesicles which stimulate healing, tissue regeneration, and/or suppress adverse immune reactions [8].

We have recently used human bone marrow (BM)-derived MSCs to educate human macrophages ex vivo as a means of generating alternatively activated macrophages that can stimulate fibroblast proliferation in vitro and are effective in a lethal xenogeneic ARS model [9]. We then showed macrophages could be educated with exosomes from BM-MSCs and that the most effective exosomes were generated from MSCs primed with *E. coli* lipopolysaccharide (LPS) in a dose-dependent fashion [10]. LPS is a classic toll-like receptor 4 (TLR-4) agonist and was selected in part due to recent work by others showing LPS stimulation generates MSC exosomes that promote wound healing in a diabetes model [11]. We observed that LPS-exosome educated macrophages prevented the lethal effects of ARS in part by promoting hematopoietic tissue recovery in the bone marrow and spleen, leading to improved complete blood counts (CBCs). The limitation of these macrophages, especially if used as an autologous fresh product, is the biomanufacturing time of 10 days, prohibitively too long considering patients need to be treated as soon as possible after radiation injury. Decreasing manufacturing time would potentially benefit future patients, as treatment could theoretically be given within 24 h post-radiation exposure.

The aim of this study is to determine if undifferentiated monocytes from human peripheral blood could be educated with LPS-primed, BM-MSC exosomes (LPS-EEMos) in order to bypass the need for the 7–10-day differentiation process to macrophages. Monocytes are also non-adherent and much easier to harvest from apheresis compared to strongly adherent macrophages from flasks. Autologous monocytes could be quickly isolated from ARS victims and educated within 24 h of radiation exposure, which would greatly increase the therapeutic potential of this cellular treatment for ARS.

## Methods

### Isolation of primary human monocytes

Human monocytes were derived from G-CSF-mobilized peripheral blood from healthy donors using an institutional review board (IRB)-approved protocol (2016-0298). Peripheral blood mononuclear cells (PBMCs) were first isolated using Ficoll-Paque Plus (endotoxin tested) (GE Healthcare Bio-Sciences, Piscataway, NJ, USA) by density gradient separation. The cells were recovered and contaminating red blood cells were lysed with ACK lysis buffer (Lonza, Walkersville, MD, USA) for 3–5 min followed by washing with phosphate-buffered saline (PBS) (Hyclone, Logan, UT, USA). Monocytes were isolated by labeling with anti-human CD14 microbeads (Miltenyi Biotec, Bergisch Gladbach, Germany) and an autoMACS Pro Separator instrument (Miltenyi Biotec) as directed by the manufacturer. The isolated cells were counted using Trypan blue, aliquoted into cryo-vials, and stored in liquid nitrogen in the vapor phase. The purity of the stored cells was verified post-thaw by flow cytometry and found to be > 85% CD14+ monocytes.

### Isolation and characterization of exosomes from LPS-primed human MSCs

MSCs were isolated from three different human BM samples from healthy donors using an IRB-approved protocol (2016-0298). The identity of the MSCs was confirmed by surface marker analysis confirmed by flow cytometry as previously described [12]. Exosomes were isolated from BM-MSCs between 4 and 8 passages. Briefly, BM-MSCs were grown in alpha MEM media (Corning CellGro, Manassas, VA) supplemented with 10% heat-inactivated fetal bovine serum (FBS) (Hyclone, Logan, UT), 100X l-Ala-l-Glutamine (Corning GlutaGro), and 100X NEAA (Corning) in T75 flasks. BM-MSCs were washed with PBS (Hyclone), and media from each T75 flask were replaced with 10 mL of MSC serum-free media (SFM) (StemPro A103332-01, Thermo Fisher Scientific, Waltham, MA, USA) at a concentration of approximately 1 × 10^5^ MSCs/mL. Cells were then incubated for 18–24 h and the conditioned SFM was collected and processed to isolate MSC exosomes. To produce exosomes from MSCs stimulated with LPS, MSCs were primed by supplementing the SFM with 1.0 μg/mL *E. coli* LPS O111:B4 (L4391 Sigma, St Louis, MO, USA) and then incubated for 18–24 h. The conditioned SFM was then collected and processed to isolate LPS-MSC exosomes.

Exosomes were isolated by a 2-step centrifugation process as described [13]. Briefly, the collected conditioned SFM was centrifuged at a low-speed spin (2000 × *g* at 4 °C for 20 min) to remove any detached cells, apoptotic bodies, and cell debris. The clarified supernatant was collected and added to 30-mL conical ultracentrifuge tubes (each tube contained approximately 30 mL of clarified conditioned media) and then was ultra-centrifuged for 2 h using Optima™ L-80XP Ultracentrifuge (Beckman Coulter) at 100,000 *g*_avg_ at 4 °C. The supernatant was carefully aspirated, and the exosome pellet in each 30-mL ultracentrifuge tube was resuspended in 100 μL of PBS and then stored at −80 °C. A sample of the exosomes in PBS was sent to Zen-Bio, Inc. (Research Triangle Park, NC, USA) for protein and RNA concentration using a NanoDrop spectrophotometer. Particle size and concentration were determined using an IZON qNano Nanoparticle Characterization instrument (IZON, Medford, MA, USA). Particle size analysis indicated extracellular vesicle material fell within the exosome size range of < 200 nm [10].

### Surface marker analysis of exosomes by MACSplex flow cytometry

Characterization of exosomes was performed by flow cytometry at Zen-Bio, Inc. using the MACSPlex Exosome Kit (Miltenyi Biotec, Bergisch Gladbach, Germany) according to the manufacturer’s protocol, which allows the detection of up to 38 known exosomal surface markers. In brief, 25 μL of either MSC exosomes or LPS-MSC exosomes were mixed with an equal volume of capture beads coupled with exosome surface marker antibodies plus two isotype controls and gently rotated in the dark at 4 °C overnight. The bead-exosome complexes were washed and then incubated for 1 h with a detection bead mixture consisting of pan-exosome markers CD9, CD63, and CD81 labeled with FITC, PE, or APC. The beads were then washed and resuspended in 150μL of MACSPlex buffer for analysis. Prior to experimentation, the system was calibrated and background settings were adjusted to unlabeled beads. The autosampler used 100 μL from each sample to collect beads and automated gating strategies were used to identify bead populations for each analyte. Batch analysis quantified median intensities for each bead population and analyte surface expression was calculated for each sample. A Miltenyi MACSQuant Analyzer 10 was utilized for sample acquisition and MACSQuantify Software v. 2.13 was used for data analysis. Median fluorescent values of 1.0 or more were considered positive and means from exosomes isolated from different MSC isolates were determined.

### Education of monocytes by exosomes

For education of monocytes using exosomes, previously purified and cryopreserved monocytes were thawed and placed in monocyte cultivation media consisting of Iscove’s modified Dulbecco’s media (Gibco Life Technologies, Grand Island, NY USA) supplemented with 10% human AB serum (Valley Biomedical, Winchester, VA, USA), 1× MEM nonessential amino acids (Mediatech, Manassas, VA, USA), 1× glutagro (Mediatech), 1× sodium pyruvate (Mediatech), and 4 μg/mL human recombinant insulin (Life Technologies, Grand Island, NY, USA). For in vitro studies such as flow cytometry or qPCR, purified CD14^+^ monocytes were plated into six-well culture plates at a concentration of 0.5–1 × 10^7^ per well. For in vivo studies, 10^7^ purified CD14^+^ monocytes were seeded into a T75 cm^2^ filter cap cell culture flask (Greiner Bio-One, Monroe, NC, USA). Monocytes were immediately treated with either PBS (control monocytes) or educated with 5 × 10^9^ exosomes from either unprimed MSC exosomes (EEMos) or LPS-primed MSCs (LPS-EEMos) at the same particle concentration. Monocytes were then incubated at 37 °C with 5% CO_2_ for either 3 days or 24 h. Monocytes were collected by gently sweeping the flask bottom using a 1.8-cm blade cell scraper (Corning Science, Tamaulipas, Mexico), transferred to a centrifuge tube, and washed with PBS by centrifugation at 300 × g for 10 min.

### Gene expression analysis of educated monocytes

RNA was purified using a RNeasy micro kit (Qiagen, Valencia, CA, USA) from primary monocytes from 3 different healthy donors. Monocytes were educated for 24 h with either exosomes from unprimed MSCs or LPS-primed MSCs to produce EEMos or LPS-EEMos, respectively. Control monocytes were treated for 24 h with PBS. The isolated RNA was checked for quality using an Epoch microplate reader (BioTeK Instrument Inc, Winooski, VT, USA), and the RNA was converted to cDNA using Verso cDNA synthesis kit (Thermo Scientific, Pittsburgh, PA, USA) and a Veriti Thermal Cycler (Applied Biosystems, Foster City, CA, USA). Quantitative polymerase chain reaction (PCR) was performed using Power SYBR green master mix (Applied Biosystems) on StepOne Plus instrument (Applied Biosystems) using standard protocols. Verified primers for indoleamine 2, 3-dioxygenase (IDO), interleukin (IL)-6, IL-8, IL-7, IL-10, IL-12, IL-15, fibroblast growth factor-2 (FGF2), epidermal growth factor (EGF), and vascular endothelial growth factor-A (VEGF-A) were purchased from Qiagen. The comparative threshold cycle method (Ct) was used to calculate mRNA levels. Ct values for the *GAPDH* housekeeping gene and the genes of interest were determined, and the difference between the Ct values of each gene of interest and the mean *GAPDH* Ct was calculated (delta Ct). Differences in the delta Ct (delta-delta Ct) of genes in EEMos and LPS-EEMos were normalized to monocyte controls. Quantitative PCR (qPCR) data is presented as fold change expression = 2^-delta,delta Ct^ of each gene in comparison with monocyte controls.

### Flow cytometric analysis of monocytes

Monocyte controls (untreated), EEMos, and LPS-EEMos were collected and counted with a Beckman Coulter Z1 Particle Counter and then 1 × 10^6^ cells were incubated with Fc block (BD Pharmingen, San Jose, CA, USA, cat#: 564220) for 10 min at room temperature. Cells were then stained at 4 °C for 20–30 min with anti-human antibodies in staining buffer (PBS with 2% FBS). All antibodies were purchased from BioLegend (San Diego, CA). Antibodies included CD206 (15-2, cat# 321105), CD163 (GHI/61, cat# 333617), PD-L1 (29E.2A3, cat# 329721), PD-L2 (24F.10C12, cat# 329608), CD14 (HCD14, cat# 325627), CD16 (3G8, cat# 302025), HLA-DR (L243, cat# 307639), CD73 (TY/11.8, cat# 127223), and CD86 (IT2.2, cat# 305431). The cells were washed with PBS, centrifuged at 300g for 10 min, and Ghost Dye™ Red 780 viability dye (Tonbo Biosciences, cat# 13-0865) was added for 20 min at room temperature. Cells were then washed with staining buffer, spun down, and resuspended in 400 μL of staining buffer. Cells were then run on an Attune™ NXT flow cytometer (Thermo Fisher Scientific). Subsequent analysis was performed using Flowjo™ 9.9.6 software (BD).

### Mice

NOD.Cg-*Prkdc*^*scid*^ *Il2rg*^*tm1Wjl*^/SzJ (NSG) mice were purchased from The Jackson Laboratory (Bar Harbor, Maine, USA). Both male and female mice were used at 8–16 weeks of age, and both sexes were used in a given ARS model experiment unless otherwise indicated. All animals were bred and housed in a pathogen-free facility throughout the study. The Animal Care and Use Committee at the University of Wisconsin-Madison approved all experimental protocols (M005915).

### Lethal acute radiation syndrome xenogeneic model

On day 0, NSG mice received 4 Gy lethal total body irradiation using an X-RAD 320 X-ray irradiator (Precision X-Ray, North Branford, CT, USA). Either 4 (4 h post), 24 (24 h post), or 48 (48 h post) hours after radiation challenge, mice were treated with a single injection in the tail vein with 100 μL of either PBS, 1 × 10^7^ control monocytes, 1 × 10^7^ EEMos, or 1 × 10^7^ LPS-EEMos. Mice were typically monitored at least 3 times per week with clinical scores and survival recorded for each mouse. Clinical scores were determined based on a modified clinical scoring system as previously described [14]. Five clinical categories were monitored and graded with a score from 0 to 2 (normal to poor) and a cumulative score was determined for each mouse. Specifically, these categories are as follows: *Weight loss*: grade 0 < 10%, grade 1 > 10 to < 25%, and grade 2 > 25%; *Posture*: grade 0 normal, grade 1 hunching noted at rest, and grade 2 severe hunching impairs movement; *Activity*, grade 0 normal, grade 1 mild to moderately decreased, grade 2 stationary unless stimulated; *Fur texture*: grade 0 normal, grade 1 mild to moderate ruffling, grade 2 severe ruffling or poor grooming; and *Skin Integrity*: grade 0 normal, grade 1 scaling of paws or tail, and grade 2 obvious areas of the exposed skin.

Complete blood counts (CBCs) were performed by nicking the tail vein and collecting peripheral blood in a microtainer K_2_ EDTA tube (cat#365974 Becton Dickenson, Franklin Lakes, NJ, USA). Whole blood was assayed on a Hemavet 950FS analyzer (Drew Scientific Inc., Miami Lakes, FL) with instrument selection for mice.

### In vivo IL-6 receptor and PD-1 inhibition

On day + 0, female or male NSG mice received 4 Gy lethal total body irradiation using an X-RAD 320 X-ray irradiator. Four hours post-radiation challenge, mice were treated with a single injection in the tail vein with 100 μL of 1 × 10^7^ LPS-EEMos or PBS. On days 2, 5, and 8 post-irradiation, the LPS-EEMo-treated mice were given intraperitoneal injections of 250 μg rat anti-mouse IL-6R (clone 15A7 from Bio X Cell, Lebanon, NH, USA), 250 μg rat anti-mouse PD-1 (clone RMP1-14, Bio X Cell), or 250 μg rat IgG2b isotype control (clone LTF-2 from Bio X Cell, Lebanon, NH, USA). Weight loss, clinical score, and survival were tracked for each mouse.

### CD34^+^ HSC viability and proliferation assays

A Ficoll density gradient centrifugation was done on G-CSF mobilized peripheral blood to isolate PBMCs. Residual red blood cells were lysed by adding 3 mL of Lonza™ BioWhittaker™ ACK lysing buffer to PBMCs at room temperature for 3 min. Cells were washed and centrifuged with PBS and then resuspended in MACS buffer (PBS with 2% FBS and 2 mM EDTA). Cells were then incubated with the CliniMACS® CD34 Reagent System for 30 min at room temperature and run on the AutoMACS® Pro separator in order to isolate CD34^+^ HSCs. CD34+ HSCs were then frozen in 90% FBS and 10% DMSO. Isolated CD34^+^ HSCs were thawed overnight in RPMI with 10% FBS, 1% penicillin-streptomycin, and 1% l-glutamine at 37^o^ C. CD34^+^ HSCs were then resuspended in StemMACS HSC expansion media XF (with the StemMACS HSC expansion cocktail added) and irradiated with 4 Gy using the Xstrahl 225 X-Ray research irradiator cabinet. CD34^+^ HSCs were then labeled with BD Horizon™ violet cell proliferation dye (VPD450). The irradiated and labeled CD34^+^ HSCs were then cultured alone or co-cultured using HSC expansion media in triplicate with LPS-EEMos, EEMos, or monocyte controls at a 1:1 ratio in a 96-well plate for 3 days. After 3 days, cells were harvested and stained with antibodies for Annexin V (Cat# 640919) and CD34 (clone 561, cat# 343615) from BioLegend® and Ghost Dye™ Red 780 viability dye from Tonbo Biosciences (cat# 13-0865). Viable cells were identified as Annexin V^−^ Ghost Dye™ Red 780^−^. Proliferation was determined by distribution of VPD450 in the CD34^+^ cell population. Both viability and proliferation were analyzed using the Attune™ NXT flow cytometer (Thermo Fisher Scientific). Subsequent analysis was performed using Flowjo™ software (BD).

## Statistical analysis

Statistics were performed using GraphPad Prism version 7.0 (GraphPad Software, San Diego, CA) and Microsoft Excel. Data were reported as mean ± SEM. Three or more groups were compared using a nonparametric analysis of variance (ANOVA) test with Dunnett’s multiple comparisons post-test performed. Mantel-Cox log-rank was used for the comparison of the Kaplan-Meier survival curve. Principal component analysis and *t* tests were performed to compare pairs of CBC data. A *p* value less than 0.05 was considered statistically significant for all tests.

## Results

### Exosomes from LPS-primed MSCs have a unique surface marker profile

**Fig. 1:**
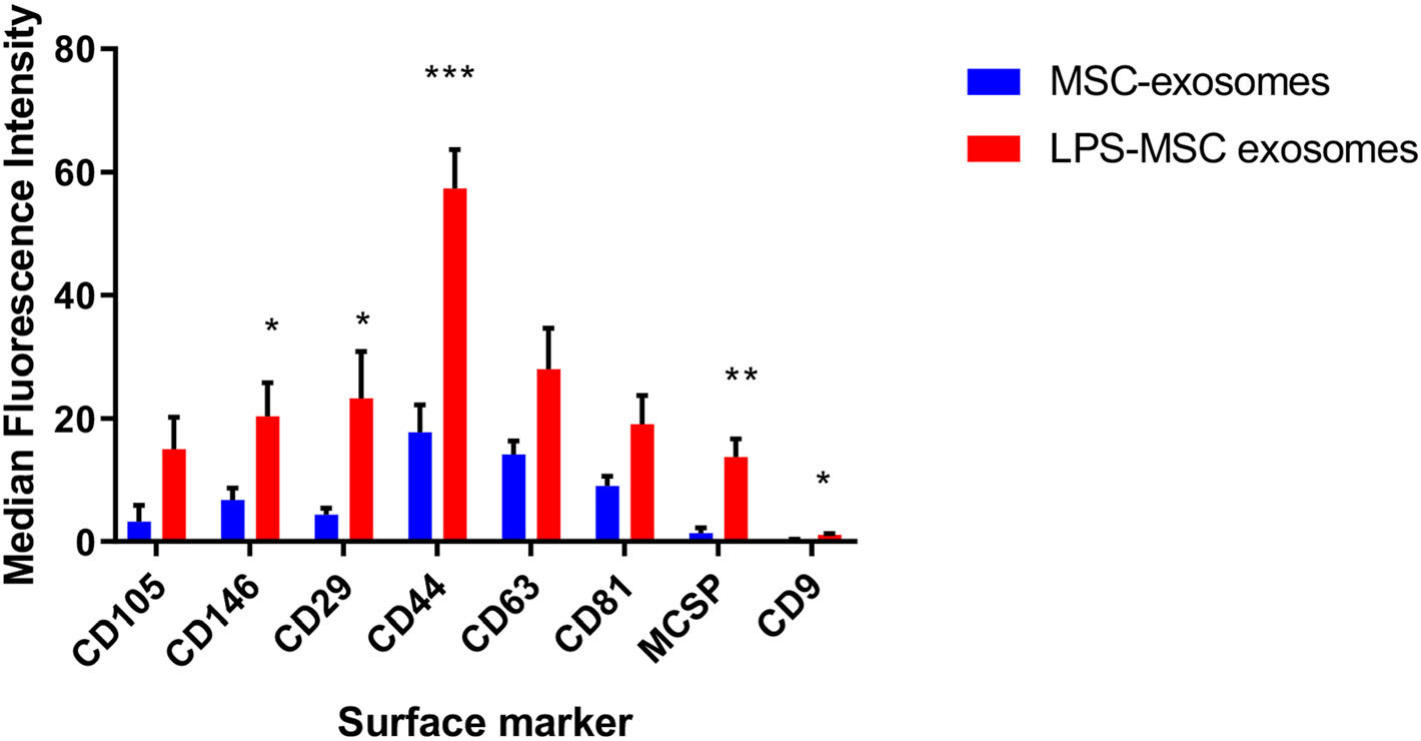
Exosomes from LPS-primed MSCs have a unique cell surface marker profile. Exosomes were isolated from BM-MSCs that were either primed with LPS (LPS-MSC exosomes) or without LPS (MSC exosomes) for 24 h. The isolated exosomes were stained overnight with 37 different bead surface marker populations and compared by mean fluorescence intensity. Results are from two replicates of two independent studies. Groups were compared by Kruskal-Wallis with a Dunn post-test. *N* = 4, *p ≤ 0.05, **p ≤ 0.005, ***p ≤ 0.0005

LPS is a ligand for TLR-4 found on the surface of MSCs and has been previously shown to augment exosome production [10, 11]. Characterization of extracellular vesicles from different human MSC isolates confirmed that unprimed MSCs and LPS-primed MSCs produced exosomes that were similar in size and quantity produced. The mean size of extracellular vesicle preparations from unprimed MSCs and LPS-primed MSCs were a typical range for exosomes, at 162 nm and 156 nm, respectively. While the mean particle concentration of LPS-primed MSC exosomes at 2.0 × 10^11^ particles/mL was higher than that of unprimed MSC exosomes at 1.2 × 10^11^ particles/mL, it was not statistically significant. However, 5 out of 37 surface markers (CD146, CD29, CD44, MCSP, and CD9) between exosomes from unprimed MSC and LPS-primed MSCs were elevated in exosomes from LPS-primed MSCs as compared to the unprimed MSCs (Fig. 1).

### LPS-EEMos have a distinct gene expression profile

**Fig. 2:**
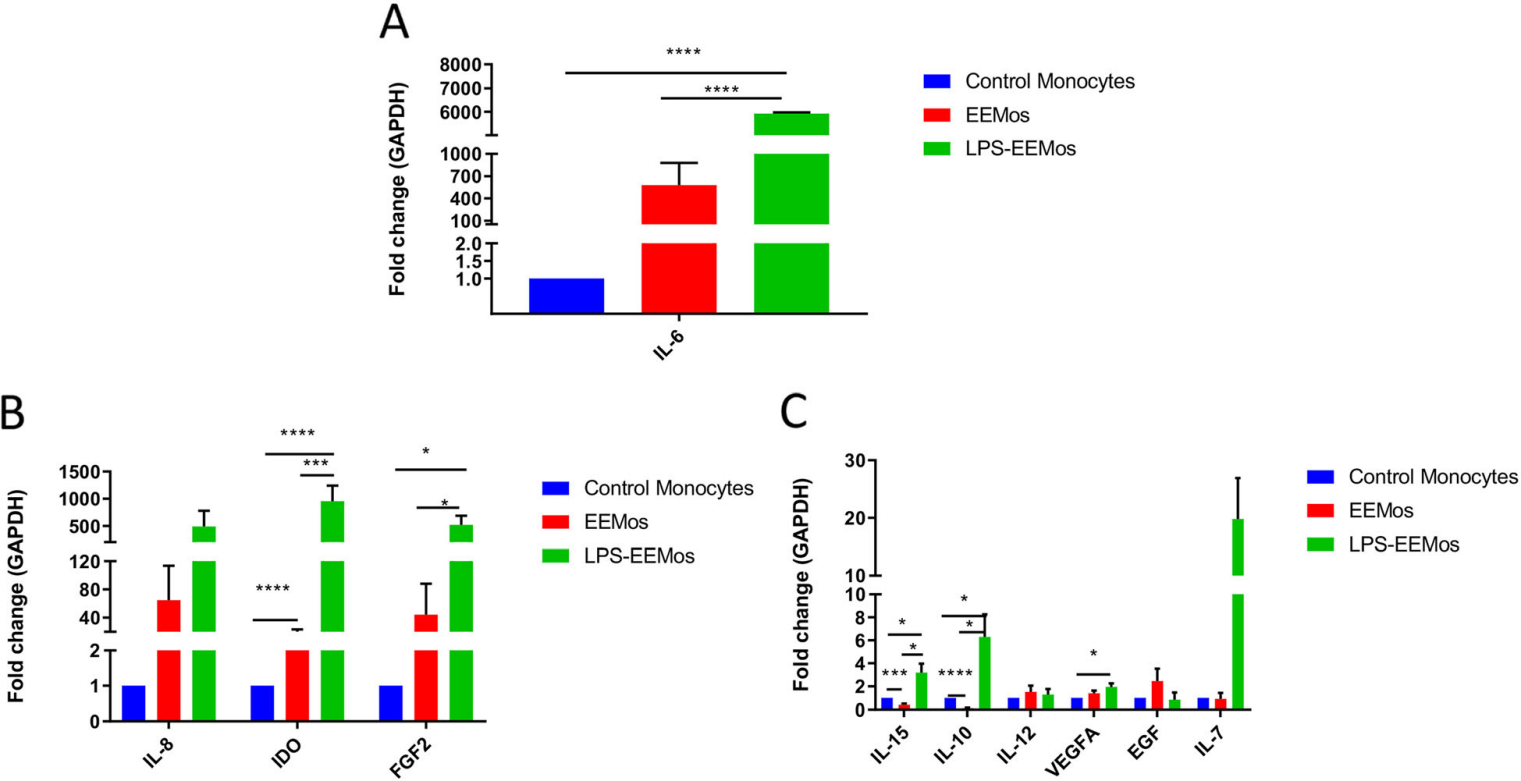
LPS-EEMos express an anti-inflammatory, immunosuppressive, and regenerative gene expression profile. Monocytes from 3 isolates were either untreated (control monocytes), treated for 24 h with unprimed MSC exosomes to produce EEMos, or with LPS-primed MSC exosomes to produce LPS-EEMos. After treatment, the cells were collected and RNA isolated and analyzed by qRT-PCR for gene expression. The fold change of gene expression normalized to a GAPDH housekeeping gene was compared to untreated control monocytes for **A** IL-6; **B** IL-8, IDO, and FGF2; and **C** IL-15, IL-10, IL-12, VEGFA, EGF, and IL-7. Results are from two replicates of three independent studies. Groups were compared by Kruskal-Wallis with a Dunn post-test. *p ≤ 0.05, ***p ≤ 0.0005, and **** *p* ≤ 0.0001 between groups are designated as compared to control monocytes

Exosomes from LPS-primed MSCs were then used to educate human monocytes (LPS-EEMos), and their gene expression of various interleukins, growth factors, and enzymes were compared to uneducated monocytes or monocytes educated with exosomes from unprimed MSCs (EEMos). LPS-EEMos showed very high gene expression of IL-6, approximately 6000-fold higher compared to control monocytes and 100-fold higher than EEMos (Fig. 2A). Gene expression of IDO and FGF2 (Fig. 2B) as well as IL-10 and IL-15 (Fig. 2C) were all higher in LPS-EEMos compared to either control monocytes or EEMos. VEGF-A gene expression was only higher in LPS-EEMos when compared to control monocytes (Fig. 2C).

### Biomanufacturing LPS-EEMos enriches a CD14^+^ PD-L1^+^ monocyte subset

**Fig. 3:**
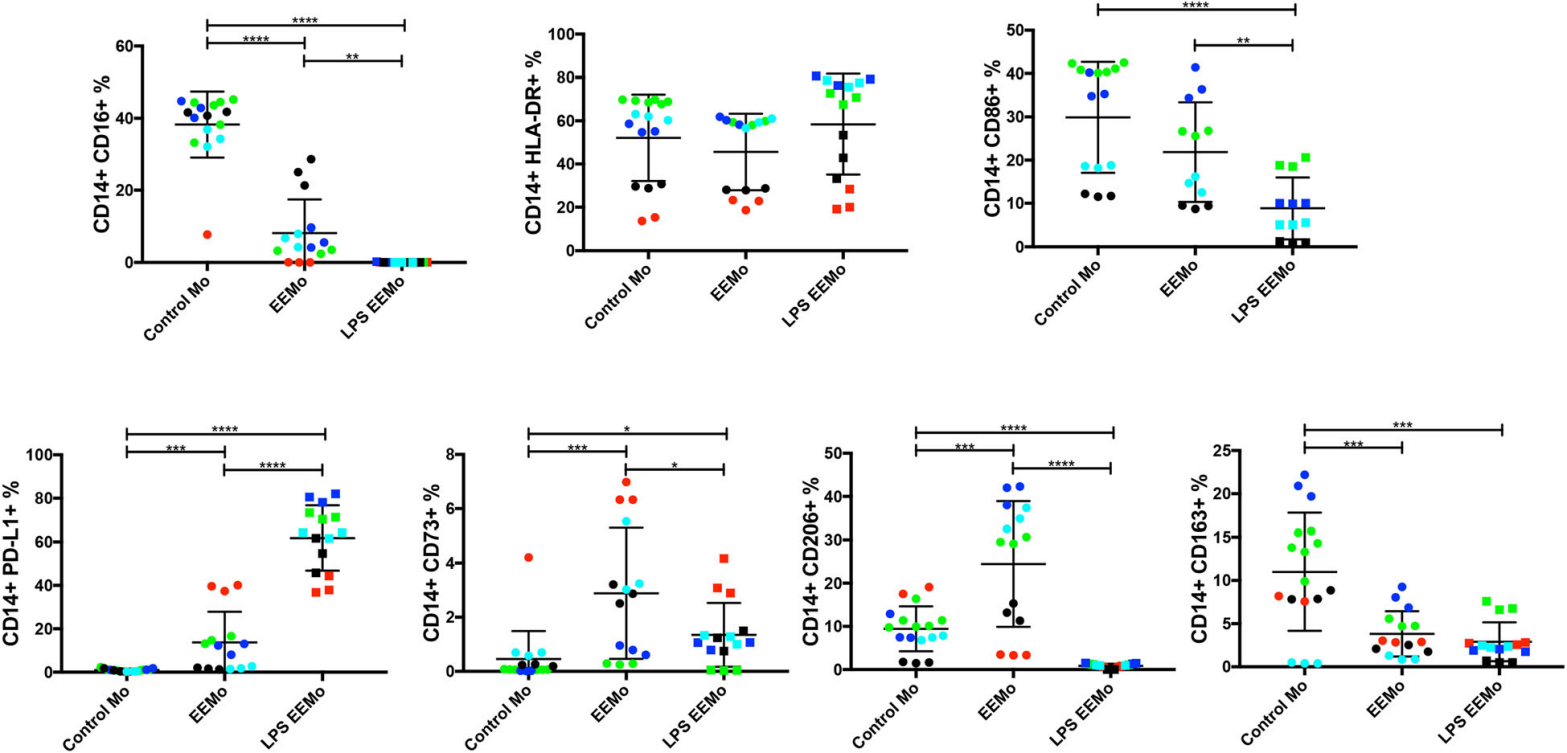
LPS-EEMos show an increased percentage of PD-L1^+^ cells and decreased percentage of CD16^+^ and CD86^+^ cells. Monocytes from 4 isolates (color coded) were either untreated control monocytes (Mo), educated for 24 h with either unprimed MSC exosomes (EEMo), or LPS-primed MSC exosomes (LPS-EEMo). After education, monocytes were stained for flow cytometry. The percent (%) CD14^+^ cells for each marker (± SEM) is shown. Results pooled from 2 separate experiments, with 4 to 13 samples/group. Groups were compared by Kruskal-Wallis with a Dunn post-test. *p ≤ 0.05, **p ≤ 0.005, ***p ≤ 0.0005, ****p ≤ 0.0001 between groups

Flow cytometric analysis showed that LPS-EEMo generation yielded distinct CD14^+^ monocyte subsets when compared to both EEMos and control monocytes. A higher percentage of PD-L1^+^ cells were enriched whereas lower percentages of CD16^+^, CD86^+^, CD73^+^, CD206^+^, and CD163^+^ cell subsets were observed (Fig. 3). PD-L2 expression was not meaningfully observed in any group (data not shown), and no differences were seen in the percentages of HLA-DR^+^ cells.

### LPS-EEMos educated for 3 days protect mice from ARS and promote hematopoietic recovery

**Fig. 4:**
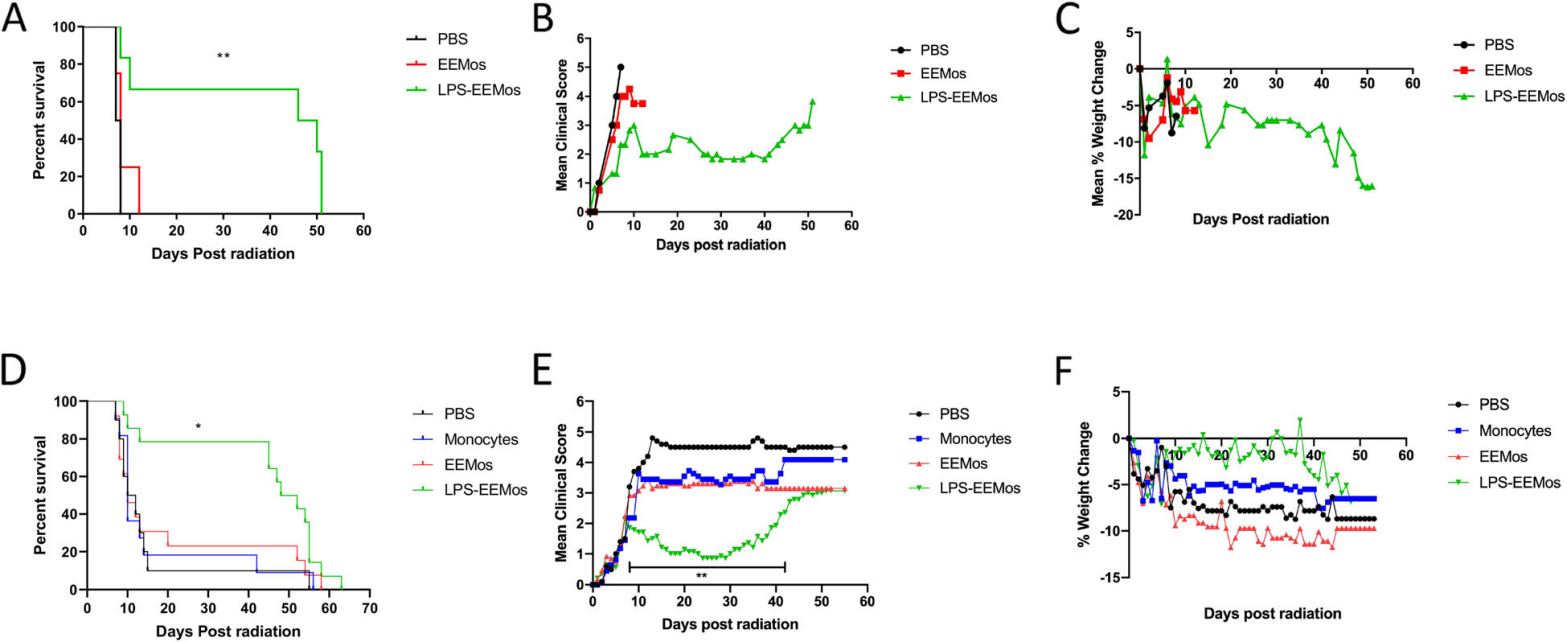
Treatment with LPS-EEMos educated for 3 days or 24 h significantly improves survival and clinical scores in mice with lethal ARS when infused 4 h after radiation exposure. On day 0, NSG mice received 4 Gy of lethal radiation followed by an i.v. treatment 4 h later with PBS (vehicle control), 1 × 10^7^ EEMos, or 1 × 10^7^ LPS-EEMos. **A** Survival curve of treated mice after radiation. **B** Mean clinical scores (percent weight loss, posture, activity, and fur texture) **C** Mean percent weight change. **D** On day 0, NSG mice received 4 Gy of lethal radiation followed by an i.v. treatment 4 h later with PBS (vehicle control), 1 × 10^7^ EEMos, or 1 × 10^7^ LPS-EEMos. Survival curve of treated mice after radiation. **E** Mean clinical scores. **F** Mean percent weight change. The final mean percent weight change and clinical score were carried over after death to allow for comparison between groups at a given time point by Kruskal-Wallis with a Dunn post-test. Results pooled from three separate experiments, with 10 to 14 mice/group, for survival analysis. A representative clinical score and weight change from one of 3 experiments are shown, with 2 to 6 mice/group. *p ≤ 0.05, **p ≤ 0.01

We established a lethal ARS xenogeneic model where a 4-Gy dose typically kills more than 50% of PBS-treated NSG mice within 10 days (Fig. 4A). These mice show a rapid increase in clinical scores (Fig. 4B) and weight loss (Fig. 4C). Complete blood counts (CBCs) nadir 4 to 6 days after radiation exposure (Table 1). LPS-EEMos were initially prepared after a 3-day education with exosomes from LPS-primed MSCs, akin to the education time we have previously used with macrophages [10], and then the educated monocytes were administered 4 h after irradiation leading to improved survival (Fig. 4A) and reduced rate of increase in clinical scores (Fig. 4B) but no impact on weight loss (Fig. 4C) compared to both PBS-treated and the 3-day educated EEMo-treated mice. The 3-day educated LPS-EEMo treatment led to a median survival time of 46 days, compared to 7 to 8 days compared with the PBS and 3-day educated EEMo-treated groups, respectively.

**Table 1:**
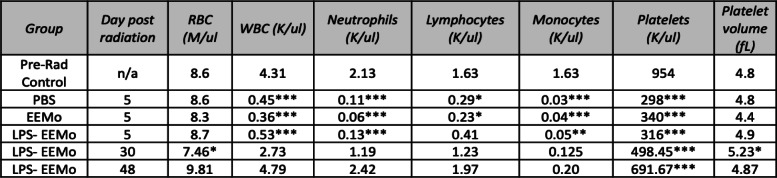
CBCs from lethally irradiated recipients of 3-day educated LPS-EEMos

Key: *n/a* not applicable. *p < 0.05, **p < 0.01, and ***p < 0.001 as compared to pre-radiation (pre-rad control). Vehicle (PBS), unprimed MSC-exosome educated monocytes (EEMo), and LPS-primed MSC-exosome educated monocytes (LPS-EEMos)

Besides ameliorating the effects of ARS, the 3-day educated LPS-EEMos promoted hematopoietic recovery as evidenced by CBCs in surviving mice. Five days after radiation exposure, all mice developed pancytopenia compared to their pre-radiation values (Table 1). On this day, 5 nadir was also seen in the LPS-EEMo-treated mice, except for the lymphocyte count which was decreased but not statistically significant. However, at day 30, the CBC in the remaining surviving mice, LPS-EEMo recipients, showed a significant and nearly full restoration to pre-radiation levels except for the red blood cell (RBC) and platelet count, but platelet volumes were higher indicating immature platelets were present. Starting at about day 40, corresponding with an increase in the clinical score (Fig. 4B), the beneficial effects of LPS-EEMo mice began to wear off and all remaining LPS-EEMo recipients began to die (Fig. 4A). Interestingly, the CBC did not nadir with day 48 values still at pre-radiation levels; the RBC count and platelet volume were well within the reported normal range for NSG mice at (7.22 ± 0.85) and (5.1 ± 0.39), respectively, and only mild thrombocytopenia remained (normal range 948 ± 280).

### Exosome education can be reduced to 24 h without sacrificing LPS-EEMo efficacy on ARS

We next evaluated if reducing the education time of monocytes from 3 days to 24 h, which is even a more practical biomanufacturing time, would impact the clinical efficacy of LPS-EEMos in the lethal ARS model. Monocytes were educated for 24 h with either exosomes from unprimed MSCs (EEMo) or exosomes from LPS-primed MSCs (LPS-EEMo) and then infused 4 h post-radiation exposure into the ARS model. Groups were compared to infusions of uneducated monocytes in addition to untreated PBS controls, with 80–90% of the PBS, monocyte, and 24-h EEMo-treated groups dying by day 20 (Fig. 4D). In contrast, 80% of the mice treated with 24-h LPS-EEMos survived more than 40 days (Fig. 4D). A significant improvement in clinical scores was seen in 24-h LPS-EEMo-treated mice from day 8 to day 42 compared to all other groups (Fig. 4E). Recipients of 24-h LPS-EEMos also maintained weight compared to other groups during the same timeframe (Fig. 4F). CBCs again nadired early (4–6 days) after radiation exposure in all 4 treatment groups compared to pre-radiation controls (Table 2). As observed with 3-day LPS-EEMos, with the exception of the platelet count by days 29–32, blood counts in the 24-h LPS-EEMos returned to pre-radiation levels. Since there was only one survivor in the PBS group at days 29–32, a statistical comparison was not possible but it is interesting to note that there was no evidence of CBC recovery in that animal. This contrasts with other groups treated with monocytes (educated or uneducated) at that time point, in which their CBCs generally returned normal levels in surviving mice.

**Table 2:**
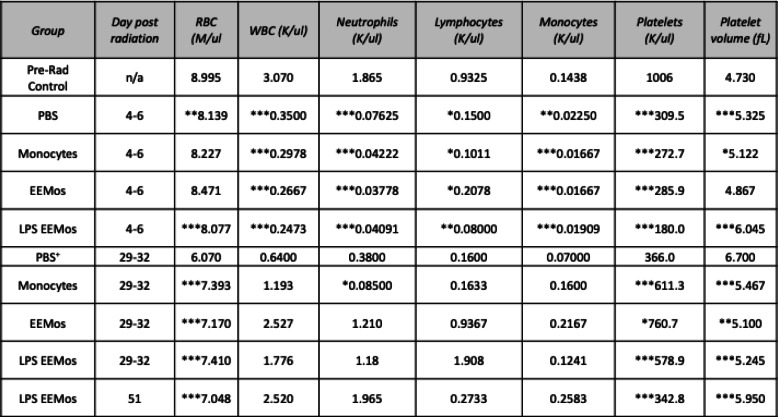
CBCs from lethally irradiated recipients of 24-h educated LPS-EEMos

Key: *n/a* not applicable. *p < 0.05, **p < 0.005, and ***p < 0.0005 as compared to pre-radiation (pre-rad control). Vehicle (PBS), uneducated monocytes (monocytes), unprimed MSC-exosome educated monocytes (EEMo), and LPS-primed MSC-exosome educated monocytes (LPS-EEMos) were each compared to pre-rad control

### IL-6 signaling is required for LPS-EEMo radioprotection from ARS lethality in female NSG mice

**Fig. 5:**
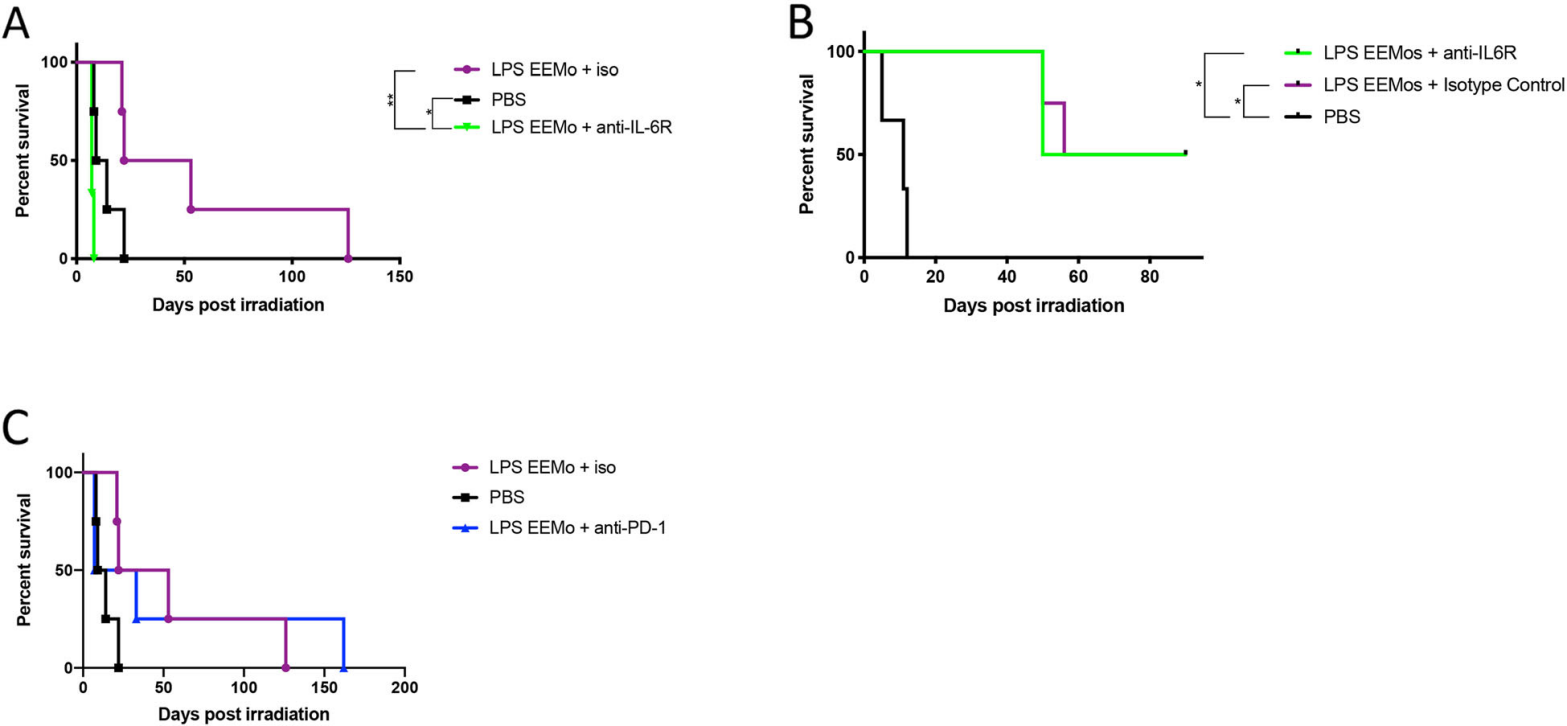
Blockade of the IL-6 receptor after lethal ARS and subsequent LPS-EEMo treatment in vivo. **A** On day 0, female NSG mice received 4 Gy lethal total body irradiation and then 4 h later were treated with LPS-EEMos (*n* = 7) or PBS (*n* = 4). On days 2, 5, and 8, LPS-EEMo-treated mice were randomized to 250 μg anti-mouse IL-6R or isotype control antibodies. **B** On day 0, male NSG mice were as treated as above with 250 μg anti-mouse IL-6R (*n* = 4) or isotype control antibodies (*n* = 4). Survival was compared by log-rank analysis. *p < 0.05, **p < 0.01. **C** On day 0, female NSG mice were treated as in A except with 250 ug anti-mouse PD-1 or isotype control antibodies (iso)

Due to prior observations of increased IL-6 production in studies of macrophages educated with exosomes from LPS-primed MSCs [10], and increased gene expression of IL-6 in LPS-EEMos (Fig. 2A), the potential mechanism of contribution by IL-6 receptor (IL6R) signaling on host tissues for radioprotection during lethal ARS was investigated in vivo. To investigate this, lethally irradiated mice were treated with LPS-EEMos and then given an anti-IL6R antibody. An antibody to the receptor was chosen rather than anti-IL6 so that impact would not depend on matching the levels of IL-6 production by LPS-EEMos, rather the idea was to saturate all available receptors on host tissues to prevent signaling from any concentration of IL-6. After IL-6R blockade in female NSG mice, the radioprotective effect of LPS-EEMos was completely abrogated (Fig. 5A), with all mice dying by day 8 post-irradiation. This survival rate was significantly lower than mice that were treated with LPS-EEMos and isotype control antibodies as well as mice that were only treated with PBS. In a repeat experiment with male NSG mice, the impact on survival was not observed (Fig. 5B); however, day 41 CBCs showed that mice treated with LPS-EEMo + anti-IL6R had significantly lower levels of erythrocytes, white blood cells (WBCs), and absolute neutrophil counts (Table 3), indicating at a minimum IL-6 contributes in part to hematopoietic recovery for both sexes.

**Table 3:** CBCs from lethally irradiated mice treated with LPS-EEMos followed by IL-6R blockade

*Group*	*Day post rad*	*RBC (M/ul)*	*WBC (K/ul)*	*Neutrophils(K/ul)*	*Lymphocytes(K/ul)*	*Monocytes* *(K/ul)*	*Platelets (K/ul)*	*Platelet volume (fL)*
LPS-EEMos	40	8.9	2.14	1.33	0.51	0.24	630	5.1
LPS-EEMos + anti-IL6R	40	5.1*	0.94*	0.36**	0.38	0.19	425	5.2
LPS-EEMos + isotype control	40	8.9	2.6	1.71	0.54	0.30	480	4.9

*P</= 0.05. **P</=0.005 as compared to untreated LPS-EEMos

Because LPS-EEMos were also enriched for PD-L1^+^ monocytes (Fig. 3), we also treated lethally irradiated mice with LPS-EEMos and then gave anti-PD1 antibody to interfere with any potential PD-1^+^ cells or tissues that would engage with LPS-EEMos. However, we did not observe any impact on survival with this intervention (Fig. 5C).

### LPS-EEMos are effective against ARS when administered 24 h post-radiation exposure

**Fig. 6:**
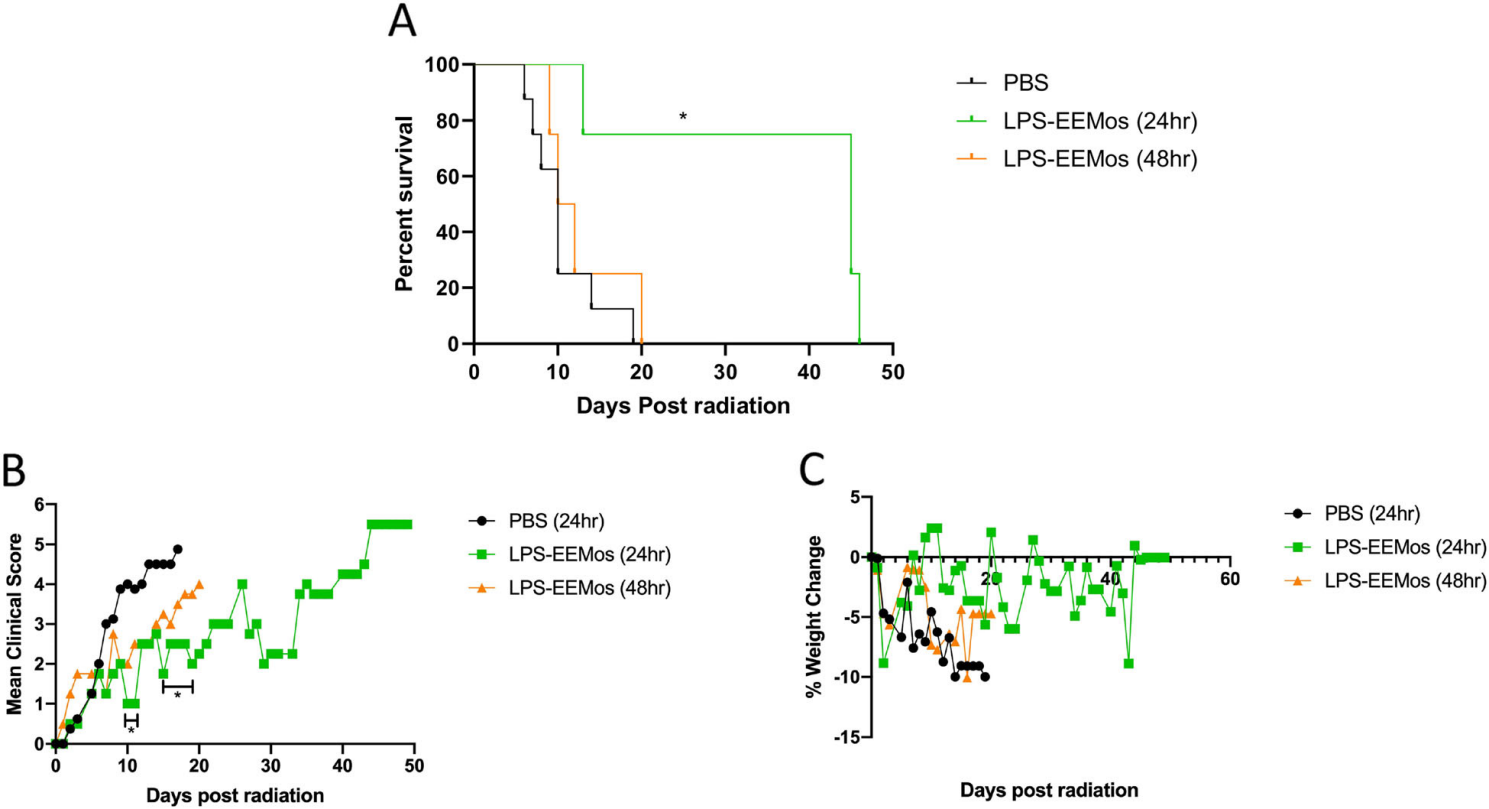
Treatment with 24-h educated LPS-EEMos treats hematopoietic ARS when infused 24 h post-radiation exposure. On day 0, NSG mice received 4 Gy of lethal radiation followed by an i.v. treatment either 24 h later with PBS (vehicle control) or 1 × 10^7^ LPS-EEMos, or 48 h later with 1 × 10^7^ LPS-EEMos. **A** Survival curve of treated mice after radiation. **B** Mean clinical scores (percent weight loss, posture, activity, and fur texture) compared to PBS controls. **C** Mean percent weight change compared with PBS controls. The final mean percent weight change and clinical score for a given animal were carried over after death to allow for **c**omparison by Kruskal-Wallis with a Dunn post-test between groups at a given time point. Results pooled from two separate experiments, with 4 to 8 mice/group, *p ≤ 0.05

While we were able to reduce the biomanufacturing time from 3 days to 24 h without losing the efficacy of LPS-EEMos, the benefits were observed when the monocytes were given 4 h after irradiation. Outside of a hospital exposure, infusions given 24 h or later after radiation exposure are more practical for clinical translation. Thus, 24-h LPS-EEMos were infused into the lethal ARS model 24 h and 48 h after radiation injury to determine if there is a therapeutic window. LPS-EEMos could significantly protect the mice from death even 24 h post-radiation challenge (Fig. 6A). Survival times (45–48 days) in these mice were similar to that seen in the 4-h post-challenge treatment results (Fig. 4A, D). While clinical scores (Fig. 6B) also significantly improved, the extent of improvement in clinical scores was not as profound when compared to 4-h post-treatment clinical scores (Fig. 4E). Infusing LPS-EEMos 48 h after radiation exposure did not protect mice from ARS lethality (Fig. 6A) or improve clinical scores (Fig. 6B). The only group to show mean weight gain at any timepoints were those mice treated with LPS-EEMos after 24 h, although there were no significant differences between groups (Fig. 6C). As seen in the 4-h post-treatment studies (Tables 1 and 2), the WBC and platelets also dropped significantly early (day 4) in mice treated 24 or 48 h post-challenge compared to pre-radiation levels (Table 4). But by day 29, a partial WBC recovery occurred in the mice treated with LPS-EEMos 24-h post-radiation exposure (Table 4), but not to the degree seen after LPS-EEMos given 4 h post-radiation (Table 1, day 30). Specifically, surviving mice who were infused 24 h post-radiation exposure showed a significant recovery in lymphocytes and monocytes, but not total WBC or absolute neutrophil counts (Table 4). As also seen after 4 h post-treatment (Tables 1 and 2), the platelet count remained significantly lower at this time point (Table 4). By day 44, mice treated LPS-EEMos 24 h after radiation injury were exhibiting clinical signs of ARS (Fig. 6B). Absolute neutrophil counts at that time were significantly higher than even the pre-radiation levels (Table 4), suggesting predisposition to secondary bacterial translocation/infection was less likely in these mice.

**Table 4:**
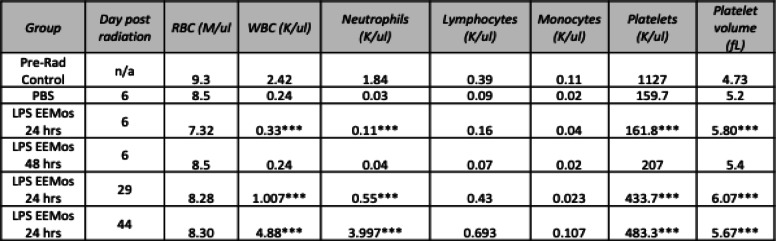
CBCs from lethally irradiated recipients of LPS-EEMos 24 and 48 h post-irradiation

Key: *n/a* not applicable. ***p < 0.0005 as compared to pre-radiation (pre-rad control). Vehicle (PBS); LPS-primed MSC-exosome educated monocytes (LPS-EEMos)

### LPS-EEMos impact the viability and proliferation of irradiated human CD34^+^ HSC progenitors in vitro

**Fig. 7:**
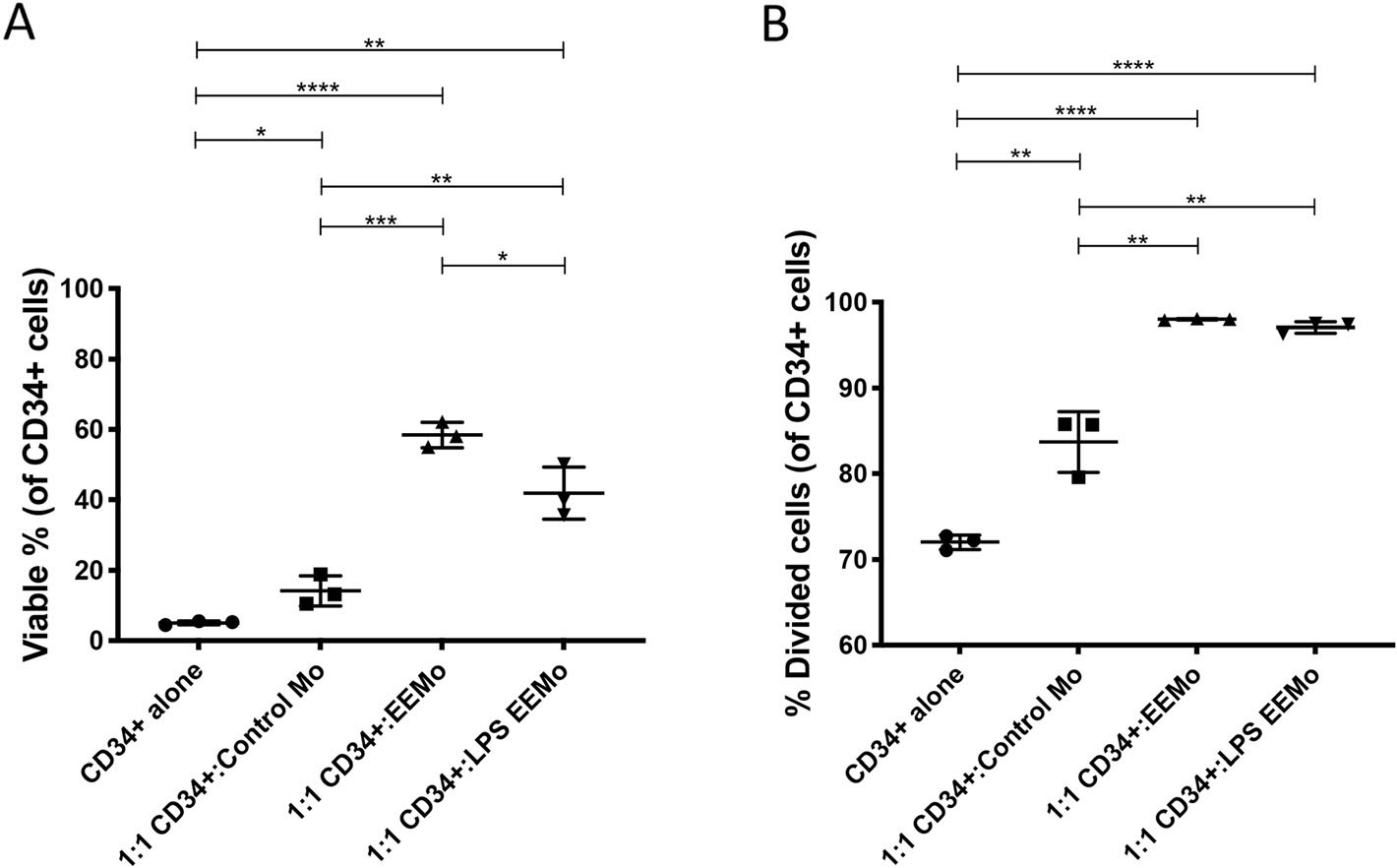
Co-culture of LPS-EEMos with irradiated human CD34^+^ HSCs enhances viability and proliferation. Cryopreserved CD34^+^ HSCs isolated from G-CSF mobilized peripheral blood were thawed and irradiated with 4 Gy and labeled with VPD450 proliferation dye. The irradiated, VPD450-labeled CD34+ HSCs were then cultured alone or co-cultured in triplicate with LPS-EEMos, EEMos, or monocyte controls at a 1:1 ratio in a 96-well plate for 3 days. After 3 days, cells were stained for **A** Annexin V, CD34, and Ghost Dye™ Red 780 viability dye. Viable cells were identified as Annexin V^−^ Ghost Dye™ Red 780^−^. **B** Proliferation was determined by distribution of VPD450 in the CD34^+^ HSC population. Results compared by Kruskal-Wallis with Dunn’s post-test. Results are representative from 2 separate experiments, each using a different monocyte isolate. *p ≤ 0.05, **p < 0.01, ***p < 0.001, ****p < 0.0001

Because the beneficial impact of LPS-EEMos in the lethal ARS model depends on a xenogeneic interaction of the educated human monocytes and/or human IL-6 on murine hematopoietic progenitors in the bone marrow, we also wanted to determine if beneficial effects can be seen with human hematopoietic progenitor cells. The viability of 4Gy irradiated human CD34^+^ HSCs isolated from G-CSF mobilized peripheral blood was more than doubled 72 h post-irradiation when co-cultured with either EEMos or LPS-EEMos compared to being cultured alone or with control monocytes (Fig. 7A). In addition, the percentage of viable CD34^+^ HSCs that had undergone at least one cell division was significantly higher when co-cultured with either EEMos or LPS-EEMos, when compared to viable CD34^+^ HSCs that were co-cultured alone or with control monocytes (Fig. 7B). These data suggest the hematopoietic recovery observed with the irradiated murine bone marrow after LPS-EEMo infusion may translate to similar benefits to hematopoietic recovery after ARS in humans.

## Discussion

The goal of this study was to develop an effective cell-based radiation counter-measure that could practically be biomanufactured and infused to treat hematopoietic ARS in a clinical setting. Cell-based radiotherapeutics need to be available in a timeframe when real-life intervention is likely after a radiation exposure. Ideally, a cell-based radiotherapeutic would either be an allogeneic, cryopreserved, “off-the-shelf” cell therapy that could be thawed after radiation exposure or be prepared fresh as an autologous product that could be available within 24 h of radiation exposure. In this study, we show that exosomes from LPS-primed MSCs can educate monocytes (LPS-EEMos) to promote hematopoiesis in vitro and in vivo, as well as function as a radiotherapeutic in a lethal ARS model, with the added benefit of reducing biomanufacturing time from 7 to 10 days to 24 h. In addition, we observed that these LPS-EEMos demonstrate a survival benefit when infused 4 h after radiation exposure in an IL-6-dependent manner, and also observed benefits when infused 24 h after radiation exposure but not after 48 h.

Except for a recent report using interferon-stimulated autologous monocytes to treat ovarian cancer [15], to our knowledge, very few reports describe the therapeutic use of monocytes as a cellular therapy. Macrophages were recently infused safely in patients with liver cirrhosis [16]. Monocytes are superior to macrophages as they can be isolated directly from peripheral blood and educated immediately, effectively eliminating the time needed for differentiation to macrophages. Monocytes circulate in the bloodstream for about 1 to 3 days and then migrate into tissues where they differentiate into macrophages. Besides being underutilized as a source of therapeutic cells, monocytes have some clear production advantages compared to macrophages. Monocytes compose of up to 10% of all leukocytes in the blood and, as opposed to macrophages, are also weakly adherent and can be harvested without the need for dissociation reagents.

We have observed that TLR-4 stimulation of MSCs through LPS priming directs them to secrete exosomes that can educate monocytes into a cell subset that promotes murine hematopoietic recovery from ARS in vivo, but also promotes human irradiated CD34^+^ HSC proliferation and survival in vitro. While we cannot rule out a synergistic effect between low levels of carryover LPS with the LPS-exosomes, the direct treatment of macrophages with LPS was ineffective in this ARS model [10]. Moreover, we observed that MSCs produced exosomes with different expression levels of 4 surface markers (CD146, CD29, CD44, and MCSP) after LPS priming. The most significant increase was in CD44, a glycoprotein receptor for hyaluronic acid and other ligands, such as osteopontin, collagens, and matrix metalloproteinases [17]. Interestingly, all of these CD44 ligands promote tissue remodeling. For example, hyaluronic acid is a main component of the extracellular matrix and induces fibroblast migration and activates to wound healing [18]. The other elevated markers, CD146, CD29, and MCSP, are involved in cell-cell/cell-matrix interactions and wound healing [19]. Overall, the interaction of these surface markers on exosomes with monocytes may improve the capacity of the educated monocytes to drive the tissue repair in ARS. It is important to note that TLR-4 stimulation by LPS is not the only method to prime MSCs and generate exosomes, as TNFα [20–22], IFNγ [22, 23], and IL-1β [24, 25] have also been shown as effective molecules for priming MSCs. A head-to-head comparison of MSC priming methodologies needs to be conducted.

LPS-EEMo gene expression indicated a cell phenotype that was more anti-inflammatory, immunosuppressive, and regenerative. IL-6 is a pleiotropic cytokine and while traditionally has been considered as a pro-inflammatory cytokine it is now known that it also has a myriad of regenerative and even anti-inflammatory properties [26]. For example, recently, it has been shown that IL-6 may be an initiating signal to polarize macrophages into a more anti-inflammatory M2 state [27, 28]. Indeed, in our model, blockade of IL-6 receptor in the irradiated animal model completely reversed the beneficial effects of LPS-EEMos on survival in female NSG mice, suggesting IL-6 is necessary for the radioprotective effect of LPS-EEMos in females. Even though no survival benefit was seen in male NSG mice, anti-IL6R-treated mice had significantly lower erythrocytes, white blood cells, and neutrophils suggesting that while there is an inconsistent impact on overall survival by sex, IL-6 signaling is needed to drive hematopoietic recovery in both sexes. In fact, IL-6 knockout mice have been shown to have decreased proliferation of bone marrow progenitor cells [29], and blockade of the IL-6 receptor exacerbates intestinal injury after focal irradiation [30]. More recently, an experiment in zebrafish has indicated that the IL-6/IL6-R axis is required for HSC generation [31]. While the effect of radiation susceptibility by sex has been surprisingly understudied, some data is available from mice and humans [32]; thus, the discrepant impact of IL-6 signaling by sex on survival after lethal ARS warrants further investigation.

The expression of IDO, FGF-2, and cytokines IL-10 and IL-15 were also elevated in LPS-EEMos. IDO has been shown to be immunosuppressive for T-cells when produced by MSCs [33] and may also be an important mechanism of immunosuppression by monocytes. FGF-2 promotes cell resistance in both irradiated normal and tumor tissues, notably through the downregulation of apoptosis [34]. It has been demonstrated that intestinal crypt radioprotection by FGF-2 is dependent on the regulation of Akt/p53 signaling [16]. Moreover, FGF-2 as an angiogenic factor has a protective effect against DNA damage for endothelial cells [35]. IL-10 is an anti-inflammatory cytokine, while IL-15 is critical for the maintenance of natural killer cells and memory T cells [36]. While the mechanism driving this unique gene expression profile is unknown, one possibility is that the abundant functional non-coding RNAs within MSC exosomes may be driving the LPS-EEMo phenotype [8]. For example, one group has found that MSC exosomes can mediate cardio-protection via miR-19a [37] while another group found that MSC exosomes with elevated miR-133b improve axonal remodeling after traumatic brain injury as compared to MSC exosomes with low miR-133b expression [38].

The three major types of monocytes are essentially characterized by the level of CD16 in relation to CD14, a pan marker for both monocytes and macrophages [39]. LPS-EEMos displayed a classical monocyte (CD14^++^ CD16^−^) anti-inflammatory phenotype [40]. In contrast to the low expression of many surface markers, PD-L1 expression was significantly elevated in LPS-EEMos. The expression of PD-L1 in monocytes could be considered immunomodulatory by inducing both IL-10 production and activating CD4^+^ Th2 cells and inhibiting CD4^+^ Th1 cell function [41]. Blockade of PD-1 did not affect the radioprotective capacity of LPS-EEMos on survival after lethal ARS; however, because lymphocytes are the main cell type that express PD-1, the role of PD-1/PD-L1 on LPS-EEMos is likely better explored in an immunocompetent mouse model. The use of an immunodeficient mouse model limits the interpretation of the impact of human LPS-EEMos on reconstitution of murine hematopoietic cells; thus, further testing to determine whether the presence of mature lymphocytes has any impact on the radioprotective effect of LPS-EEMos should be further explored in ARS models using immunocompetent mice as well as with humanized mouse models to determine the impact of LPS-EEMos on human hematopoietic cell recovery. In addition, even though LPS-EEMos are washed prior to infusion, the clinical benefits could be in part from adoptively transferred exosomes from LPS-primed MSCs. Studies examining direct injection of exosomes from LPS-primed MSCs will be needed.

Benchmark guidelines recommend that radiotherapeutics should be effective at least 24 h post-radiation exposure [42]. Here, we found that the LPS-EEMos were effective at both 4 h and 24 h after radiation injury, and while were ineffective when administered 48 h post-radiation injury, still confirms this therapeutic modality when given within a certain window in the post-radiation phase. In our experiments, we observed there was a significant restoration and maintenance of blood cells, especially the WBC and absolute neutrophil count, in mice infused with LPS-EEMos after radiation exposure. We suspect there is a limited window of time to counteract or reverse damage to HSCs, but further time course studies will be needed as well as formal in vivo analysis of trafficking by LPS-EEMos to see if homing to the bone marrow is required. Lastly, studies will be needed to determine if LPS-EEMos must be generated as a fresh, autologous product, with the potential risk that the patient’s monocytes will be too damaged from radiation to be educated. Alternatively, LPS-EEMos could be generated as a cryopreserved, allogeneic product, with the potential risk of rejection by radio-resistant NK cells. With either product source, serial dosing will likely be needed since survival in the ARS model was prolonged but not durable.

## Conclusions

In summary, we show that exosomes from LPS-primed MSCs can educate monocytes into LPS-EEMos, a radio-mitigator cell subset capable of effectively reducing damage from lethal ARS, in part through production of IL-6, by stimulating hematopoietic recovery. Development of cellular therapies remains a promising area of research for managing accidental or intentional exposure to agents that cause hematopoietic ARS, and warrants continued support by governmental agencies worldwide for purposes of medical therapeutics and biodefense counter-measures.

## Data Availability

The datasets used and/or analyzed during the current study are available from the corresponding author on reasonable request.

## Abbreviations

ARS: Acute radiation syndrome
BM: Bone marrow
CBCs: Complete blood counts
Ct: Comparative threshold
EEMos: Unprimed MSC-exosome educated monocytes
EGF: Epidermal growth factor
FBS: Fetal bovine serum
FDA: Food and Drug Administration
FGF2: Fibroblast growth factor-2
HSC: Hematopoietic stem cell
IDO: Indoleamine 2, 3-dioxygenase
IL: Interleukin
IL-6R: Interleukin-6 receptor
IRB: Institutional review board
LPS: Lipopolysaccharide
LPS-EEMos: Lipopolysaccharide-primed, MSC-exosome educated monocytes
MSCs: Mesenchymal stromal cells
NSG: NOD-SCIDγc^−/−^
PBMCs: Peripheral blood mononuclear cells
PBS: Phosphate-buffered saline
PCR: Polymerase chain reaction
qPCR: Quantitative polymerase chain reaction
RBC: Red blood cell
SFM: Serum-free media
TLR-4: Toll-like receptor 4
VEGF-A: Vascular endothelial growth factor-A
WBC: White blood cell
